# Global survey of women’s awareness of pregnancy- and postpartum-associated venous thromboembolism (World Thrombosis Day 2025)

**DOI:** 10.1016/j.rpth.2026.103421

**Published:** 2026-03-23

**Authors:** Marcello Di Nisio, Isabelle Mahé, Parham Sadeghipour, John Suviraj, Anila Rashid, Ann Marie O’Neill, Beverley J. Hunt, Sabrina Hadim, Fahimeh Ghotbizadeh, Ettore Porreca, Helen Okoye, Marco Liberati, Jaromir Gumulec, Saskia Middeldorp, Cihan Ay, Cedric Hermans, Manuel Monreal, Patricia Casais, Nicola Potere, Matteo Candeloro, Lana A. Castellucci, Jean M. Connors, Aaron M. Wendelboe, Danny Hsu, Lai Heng Lee, Soo-Mee Bang, Eriko Morishita, Pantep Angchaisuksiri, Daniel Duerschmied, Benedetta Madaro, Leonie Rimann, Cary R. Clark, Clarence Potter, Erich Vinicius De Paula, Stefano Barco

**Affiliations:** 1 Department of Medicine and Ageing Sciences, University G. D’Annunzio Chieti–Pescara, Chieti, Italy; 2 Université Paris Cité, Internal Medicine Department, Assistance Publique des Hôpitaux de Paris, Louis Mourier, Hospital, Inserm UMR-S970 Paris, Cardiovascular Research Center, Team Endotheliopathy and Hemostasis Disorders, Paris, France; 3 Vascular Disease and Thrombosis Research Center, Rajaie Cardiovascular Institute, Tehran, Iran; 4 Venous Thrombo Embolism Prevention & Control Committee, Sir Ganga Ram Hospital, New Delhi, India; 5 King’s Mill Hospital, Sherwood Forest NHS Foundation Trust, Sutton-in-Ashfield, United Kingdom; 6 Thrombosis Ireland, Dublin, Ireland; 7 Guy’s and St Thomas’ NHS Foundation Trust, London, United Kingdom; 8 Imam Khomeini Hospital, Tehran University of Medical Sciences, Tehran, Iran; 9 Department of Innovative Technologies in Medicine and Dentistry, School of Medicine and Health Sciences, University G. D’Annunzio Chieti–Pescara, Chieti, Italy; 10 Department of Haematology and Immunology, College of Medicine, University of Nigeria Ituku-Ozalla, Enugu, Nigeria; 11 Obstetrics-Gynecology Clinic, SS. Annunziata Hospital, University G. D’Annunzio Chieti–Pescara, Chieti, Italy; 12 Department of Hematooncology, University Hospital Ostrava, Ostrava, Czech Republic; 13 Department of Hematooncology, Faculty of Medicine, University of Ostrava, Ostrava, Czech Republic; 14 Department of Internal Medicine, Radboud University Medical Center, Nijmegen, The Netherlands; 15 Division of Haematology and Haemostaseology, Department of Medicine I, Comprehensive Cancer Center Vienna, Medical University of Vienna, Vienna, Austria; 16 Hemostasis and Thrombosis Unit, Division of Hematology, Saint-Luc University Hospital, Université catholique de Louvain, Brussels, Belgium; 17 Faculty of Health Sciences, Universidad Católica San Antonio de Murcia, Murcia, Spain; 18 Public Health Research Institute, University of Buenos Aires and Bernardino Rivadavia General Hospital, Buenos Aires, Argentina; 19 Department of Medicine, Ottawa Hospital Research Institute, University of Ottawa, Ottawa, Ontario, Canada; 20 Hematology Division, Dana Farber Cancer Institute, Brigham and Women's Hospital, Boston, Massachusetts, USA; 21 Department of Epidemiology, Fay Boozman College of Public Health, University of Arkansas for Medical Sciences, Little Rock, Arkansas, USA; 22 Haematology Department, Liverpool Hospital, Liverpool, Sydney, New South Wales, Australia; 23 Department of Hematology, Singapore General Hospital, Singapore; 24 Duke-NUS Medical School, National University of Singapore, Singapore; 25 Division of Hematology and Medical Oncology, Department of Internal Medicine, Seoul National University Bundang Hospital, Seoul National University College of Medicine, Seongnam, Republic of Korea; 26 Division of Health Sciences, Department of Clinical Laboratory Science, Graduate School of Medical Science, Kanazawa University, Kanazawa, Ishikawa, Japan; 27 Department of Hematology, Kanazawa University Hospital, Kanazawa, Ishikawa, Japan; 28 Division of Hematology, Department of Medicine, Ramathibodi Hospital, Mahidol University, Bangkok, Thailand; 29 Department of Cardiology, Haemostaseology, and Medical Intensive Care, Medical Faculty Mannheim, University Medical Centre Mannheim, German Centre for Cardiovascular Research Partner Site Heidelberg/Mannheim, Heidelberg University, Mannheim, Germany; 30 Internal Medicine Residency Program, School of Medicine, University of Insubria, Varese and Como, Italy; 31 Department of Vascular Medicine, University Hospital Zurich, Zurich, Switzerland; 32 University of Zurich, Zurich, Switzerland; 33 Programs and Education, International Society on Thrombosis and Haemostasis, Carrboro, North Carolina, USA; 34 University of Campinas School of Medicine, University of Campinas, Campinas, São Paulo, Brazil; 35 Center for Thrombosis and Hemostasis, Academic Medical Center of the Johannes Gutenberg University Mainz, Mainz, Germany

**Keywords:** awareness, education, postpartum period, pregnancy, venous thromboembolism

## Abstract

**Background:**

Venous thromboembolism (VTE) is a leading cause of maternal morbidity and mortality, yet women’s awareness during pregnancy and postpartum is not well characterized.

**Objectives:**

To assess education and awareness of pregnancy-associated VTE across countries and healthcare systems, including sources of information, perceived understanding, and experiences with anticoagulation.

**Methods:**

We conducted a cross-sectional online survey (15 languages), endorsed by the World Thrombosis Day campaign, of females who were pregnant or ≤12 months postpartum (October 2024 and September 2025). Descriptive analyses and subgroup comparisons were performed.

**Results:**

Of 3043 responses, 3025 were analyzed. Overall, 69.8% reported no VTE education during pregnancy or the postpartum period, and 1.1% received education only at diagnosis, resulting in 70.9% with no or delayed VTE education. Upon delivery, comprehension was limited, with 36.2%, 46.8%, and 9.2% of females reporting full, partial, and poor understanding, respectively. Only 10.9% of females received instructions on VTE manifestations; among these, 16.3% were not advised to seek urgent care for suspected VTE events. Knowledge of pulmonary embolism-related signs was particularly poor. Prior VTE was reported in 13.3%, and 21.4% had ever used anticoagulants. Among participants who had discussed thromboprophylaxis and/or received anticoagulants, 32.4% were unaware of bleeding risks. Approximately 20% of those informed reported psychological distress related to VTE information. Education rates differed by age, education level, ethnicity, region, and reproductive factors.

**Conclusion:**

VTE education during pregnancy and postpartum is infrequent and unevenly distributed. Enhanced educational interventions embedded within antenatal and postnatal care pathways are urgently needed to improve VTE awareness, symptom recognition, and informed decision-making.

## Essentials

• VTE is a leading cause of maternal morbidity and mortality.
• A global survey assessed VTE awareness among 3025 pregnant or postpartum women.
• Approximately 70% of participants reported receiving no education about VTE during pregnancy.
• Knowledge of VTE symptoms was poor, highlighting the need for improved patient education.

## Graphical abstract

**Figure:**
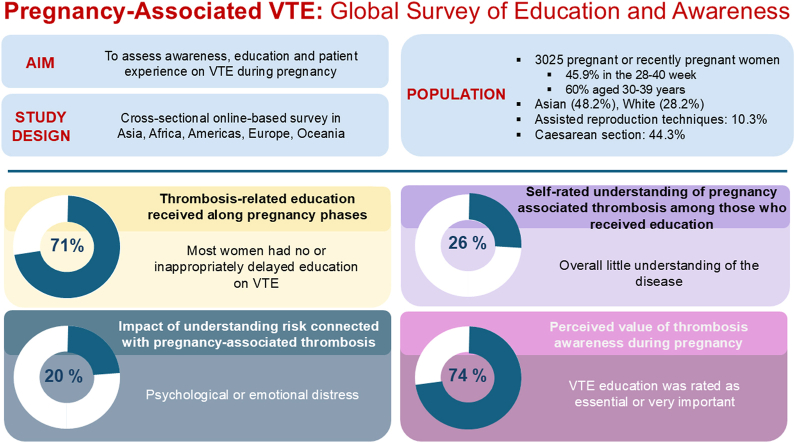

## 1 Introduction

Venous thromboembolism (VTE) encompasses deep vein thrombosis (DVT) and pulmonary embolism (PE); it is a major cause of maternal mortality and morbidity, accounting for up to 15% of all maternal deaths worldwide [1]. Beyond the acute event, VTE carries long-term consequences, including postthrombotic syndrome, functional limitations, reduced quality of life, and psychological distress [2].

Pregnancy and the postpartum period confer a 5- to 10-fold higher VTE risk compared with age-matched nonpregnant females, with an incidence of 1 to 2 per 1000 pregnancies [3,4]. Although the absolute risk of VTE is lower compared with hospitalized medical patients, the global burden is considerable given the large number of pregnancies worldwide [5]. Risk peaks during the first 6 weeks postpartum, with approximately one-third of events occurring after delivery, and returns to baseline by 12 weeks. Overall, 75% to 80% of pregnancy-associated VTE present as DVT and 20% to 25% as PE [6].

The elevated risk results from both maternal characteristics—such as previous VTE, inherited thrombophilia, obesity, and immobility—and physiological changes during pregnancy, including hypercoagulability, venous stasis, and mechanical compression of pelvic veins. Antepartum and peripartum factors, such as hyperemesis gravidarum [7], cesarean section, preeclampsia, placental complications, and infection, further increase the risk of VTE [[8], [9], [10], [11]].

Thromboprophylaxis is considered for females with prior unprovoked or hormone-related VTE, but guideline recommendations remain inconsistent due to limited and low-quality evidence [[12], [13], [14], [15], [16], [17], [18]]. Despite its incidence and impact, VTE often receives less priority than other obstetric complications, leading to low awareness among pregnant individuals, delays in diagnosis, and limited participation in preventive strategies and clinical trials. Strikingly, little is known about pregnant individuals’ knowledge of VTE during pregnancy and postpartum [19]. Therefore, this survey, endorsed by the World Thrombosis Day campaign of the International Society on Thrombosis and Haemostasis (ISTH), aimed to evaluate education and awareness of pregnancy-associated VTE across diverse countries and healthcare systems.

## 2 Methods

In this cross-sectional study, we conducted an online survey covering multiple domains related to pregnancy-associated VTE (Supplementary Table S1). The survey was intended for females who are or have been pregnant in the last 12 months. The survey questions were selected from relevant VTE domains identified in previous studies and discussions among expert physicians [20,21]. The survey was developed using a simple structure and lay language, with the goal of minimizing potential gender-, cultural-, educational-, and language-related barriers to enable broad dissemination and inclusion. A patient representative reviewed the survey draft to ensure adequacy and comprehensibility of the questionnaire format and its contents. The survey, endorsed by the World Thrombosis Day Steering Committee, was translated from English into 15 languages by native-speaking experts and made available on Research Electronic Data Capture (Vanderbilt University). For privacy and safety reasons, all data were encrypted, password-protected, and hosted on ISTH servers. Promotion and dissemination of the survey occurred primarily through social media, email newsletters, and websites. The study was considered exempt from ethics committee review, as respondents freely and anonymously completed the online questionnaire; identity could not be determined from the collected information. The survey was launched in October 2024 and closed in September 2025.

The survey collected baseline data on age, race/ethnicity, country, general education, pregnancy status, exposure to assisted reproductive techniques, and type of delivery (ie, vaginal vs cesarean). Gender identity was not collected; therefore, we use the terms “participants/respondents” or “pregnant/postpartum individuals” throughout and acknowledge the limited inference regarding sex/gender constructs. The survey explored participants’ reported education on VTE. This included the sources (eg, gynecologist/obstetrician or family doctor) and timing of education (eg, before pregnancy, during pregnancy, or at the time of blood clot diagnosis); the participant-reported understanding of the information received; VTE predisposing factors; knowledge and recognition of possible clinical manifestations of VTE; experience with anticoagulant drugs; information received about bleeding risk with anticoagulation or aspirin; and perceived value and psychological impact of education about thrombosis.

Data were presented descriptively as means, percentages, and IQRs, as appropriate. Responders were stratified by age, race/ethnicity, country, general education, pregnancy status, exposure to assisted reproductive techniques, and type of delivery. Pearson’s chi-squared test and Fisher’s exact test were used to explore potential differences across subgroups. All *P* values were from 2-sided tests, and results were deemed statistically significant at *P* < .05. All analyses were performed using JASP software and R version 4.5.0 (R Foundation for Statistical Computing).

## 3 Results

A total of 3043 questionnaires were returned, of which 18 (0.5%) were null, empty, or incomplete, resulting in more than 90% missing values. The population included in the final analysis comprised 3025 responders.

Table 1 shows the main characteristics of the survey population. The majority were aged 30 to 39 years (59.7%). With respect to self-reported ethnicity, the largest group was Asian (48.2%), followed by White (28.2%), Black or African American (10.6%), and Hispanic/Latino (1.7%). Responders resided in 38 countries or territories across Europe (49.8%), Asia (48.1%), the Americas (0.9%), Africa (0.8%), and Oceania (0.4%). Regarding the level of education attained, 24.1% reported having a university degree, 25% held an advanced graduate or professional degree, and 1.9% reported no formal school education.

**Table 1:** Main baseline characteristics of the survey participants.

Survey item	Survey participants (*N* = 3025), *n* (%)^a^
Age (y)	
≤19	46 (1.5)
20-29	898 (29.7)
30-39	1805 (59.7)
≥40	276 (9.1)
Self-reported ethnicity	
Asian	1457 (48.2)
Black or African American	321 (10.6)
Hispanic/Latino	52 (1.7)
White	853 (28.2)
Other	245 (8.1)
Prefer not to say	96 (3.2)
Continent of residence	
Africa	25 (0.8)
The Americas	27 (0.9)
Asia	1456 (48.1)
Europe	1506 (49.8)
Oceania	11 (0.4)
Self-reported educational status^b^	
None	57 (1.9)
Primary school	166 (5.5)
Secondary school	707 (23.4)
College	505 (16.7)
University	729 (24.1)
Advanced graduate/professional degree	756 (25)
Other	105 (3.4)
Pregnancy status (wk)	
≤13	305 (10.1)
14-27	971 (32.1)
28-40	1387 (45.9)
≥40	86 (2.8)
Gave birth in the last 6-12 wk	182 (6)
Other	94 (3.1)
Method of conception	
Naturally	2629 (86.9)
Assisted reproduction treatment	311 (10.3)
Uncertain/prefer not to say	85 (2.8)
Mode of delivery	
Cesarean section	881 (29.1)
Vaginal delivery	1341 (44.3)
Uncertain	776 (25.6)

a Percentages are based on the total number of patients; totals may not equal 100% because of missing values.

b Highest level of school education attained.

Among 2749 respondents who reported being pregnant at the time of survey completion, 10.1% were <13 weeks pregnant, and 32.1%, 45.9%, and 2.8% were 14 to 27 weeks, 28 to 40 weeks, and >40 weeks pregnant, respectively. An additional 6% gave birth in the 6 to 12 weeks preceding the survey’s completion. Conception through assisted reproductive techniques was reported by 10.3%. Planned or actual mode of delivery was vaginal in 44.3% and cesarean section in 29.1% of the cases.

### 3.1 Prevalence, timing, and sources of VTE education

**Figure 1:**
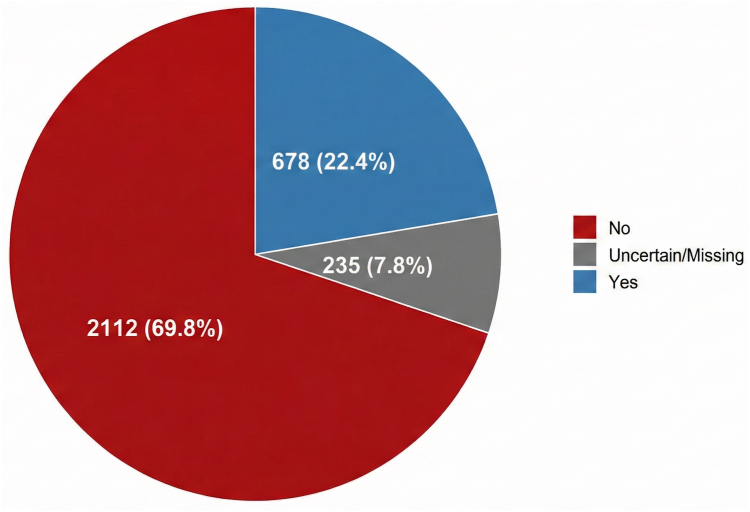
Participant-reported venous thromboembolism education received.

**Figure 2:**
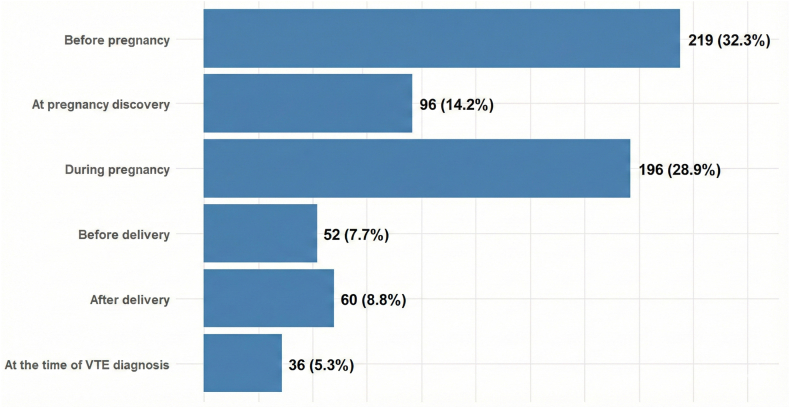
Timing of venous thromboembolism (VTE) education. Numbers refer to pregnant individuals who received VTE education.

A total of 2112 (69.8%) surveyed respondents reported receiving no education about VTE, and 36 (1.1%) received it only at the time of VTE diagnosis, resulting in 70.9% receiving no or delayed education (Figure 1 and graphical abstract). Among those who reported receiving VTE education, 20% had a history of VTE. Education occurred before pregnancy diagnosis in 32.3% of respondents, at pregnancy discovery in 14.2%, and during pregnancy in 28.9% (Figure 2).

**Figure 3:**
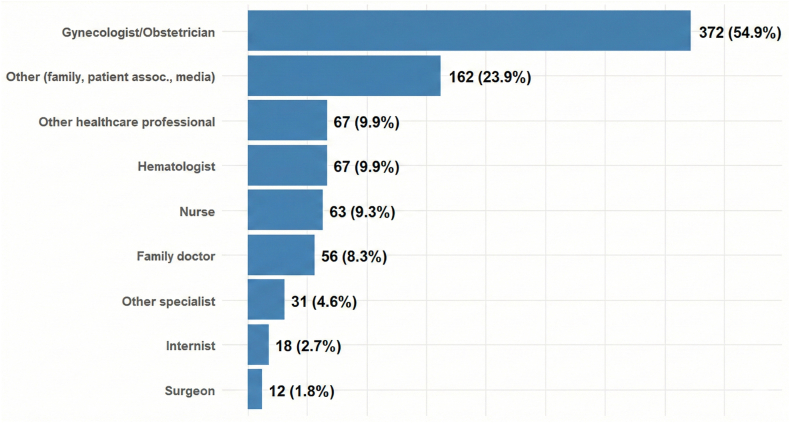
Sources of venous thromboembolism education. Numbers refer to pregnant individuals who received venous thromboembolism education.

The most frequently identified sources of VTE education were gynecologists or obstetricians (54.9%), hematologists (9.9%), nurses (9.2%), family doctors (8.2%), and other health care professionals (9.9%). The receipt of education through modalities other than direct interaction with healthcare professionals was reported by 23.9% of participants (Figure 3).

**Figure 4:**
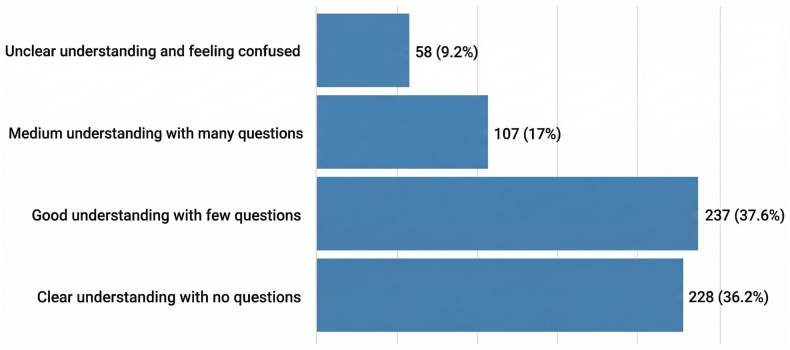
Participant-reported understanding of venous thromboembolism risk information. Numbers refer to pregnant individuals who received venous thromboembolism education.

Overall, 36.2% of respondents reported that the information was clear and required no further questions; 37.6% had a good understanding and needed a few additional questions; 17% still had many questions; and 9.2% reported a poor understanding and felt confused after receiving the information (Figure 4).

The likelihood of having received education on VTE differed significantly across respondent subgroups. Older participants were more frequently informed than younger participants, ranging from 6.5% among those aged <19 years to nearly 30% among those aged >40 years. Marked differences were also observed in self-reported ethnicity, with lower proportions of Asian participants (15.5%) reporting VTE education compared with higher education rates among White and Hispanic/Latino respondents (35.3% and 35.3%, respectively). Consistent findings were observed across geographic regions of residence. Participants with higher levels of education attained were more likely to report having received VTE education. Respondents who conceived through assisted reproduction treatment were more frequently informed than those who conceived through natural conception. Similarly, respondents who delivered by cesarean section were more likely to report VTE education compared with those delivering through vaginal delivery (Table 2).

**Table 2:** Self-reported education received by participant subgroup.

Characteristics	VTE education received, *n* (%)^a^			*P* value
	Yes	No	Uncertain	
Age (y)				
≤19	3 (6.5)	40 (87)	3 (6.5)	.002
20-29	167 (19.3)	660 (76.2)	39 (4.5)	
30-39	434 (24.8)	1233 (70.4)	85 (4.8)	
≥40	74 (27.8)	179 (67.3)	13 (4.9)	
Self-reported ethnicity				
Asian	220 (15.5)	1152 (81)	50 (3.5)	<.001
Black or African American	71 (23.2)	219 (71.6)	16 (5.2)	
Hispanic/Latino	17 (35.3)	27 (56.2)	4 (8.3)	
White	292 (35.3)	489 (59.2)	45 (5.4)	
Other	20 (21.1)	61 (64.2)	14 (14.7)	
Prefer not to say	58 (25)	163 (70.3)	11 (4.7)	
Continent of residence				
Africa	13 (52)	10 (40)	2 (8)	<.001
The Americas	10 (38.5)	14 (53.8)	2 (7.7)	
Asia	219 (15.4)	1153 (81.1)	49 (3.5)	
Europe	429 (29.6)	932 (64.4)	86 (5.9)	
Oceania	7 (63.6)	3 (27.3)	1 (9.1)	
Self-reported educational status				
None	8 (14.3)	47 (83.9)	1 (1.8)	<.001
Primary school	15 (9.4)	136 (85)	9 (5.6)	
Secondary school	123 (18.1)	528 (77.8)	28 (4.1)	
College	105 (21.4)	359 (73.1)	27 (5.5)	
University	187 (26.4)	487 (68.9)	33 (4.7)	
Advanced graduate/professional degree	212 (28.8)	487 (66.3)	36 (4.9)	
Other	28 (27.5)	68 (66.7)	6 (5.9)	
Method of conception				
Naturally	567 (22.3)	1867 (73.3)	112 (4.4)	<.001
Assisted reproduction treatment	94 (31.1)	190 (62.9)	18 (6)	
Uncertain/prefer not to say	17 (20.7)	55 (67.1)	10 (12.2)	
Mode of delivery				
Cesarean section	244 (28)	592 (68)	34 (3.9)	<.001
Vaginal delivery	274 (21.3)	961 (74.6)	53 (4.1)	
Uncertain	154 (20.5)	548 (72.9)	50 (6.5)	

VTE, venous thromboembolism.

a Due to missing values, the effective *N* for this analysis is 2930.

### 3.2 Understanding of VTE risk factors and clinical manifestations

**Figure 5:**
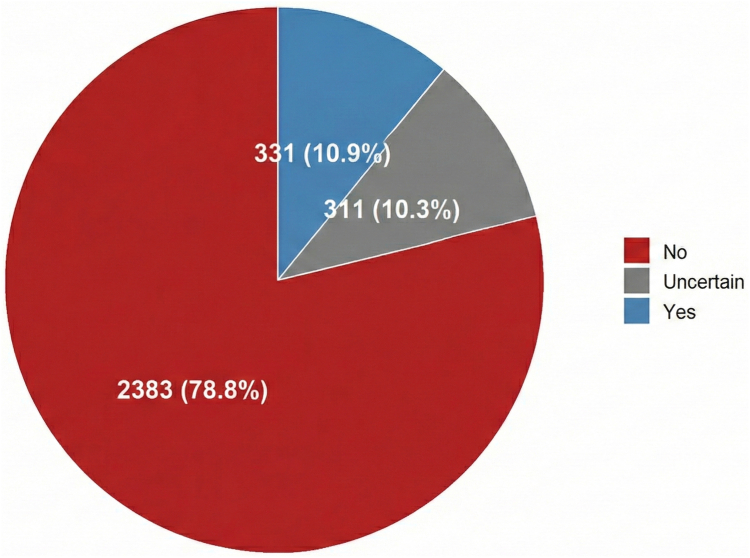
Instruction received for venous thromboembolism recognition.

**Figure 6:**
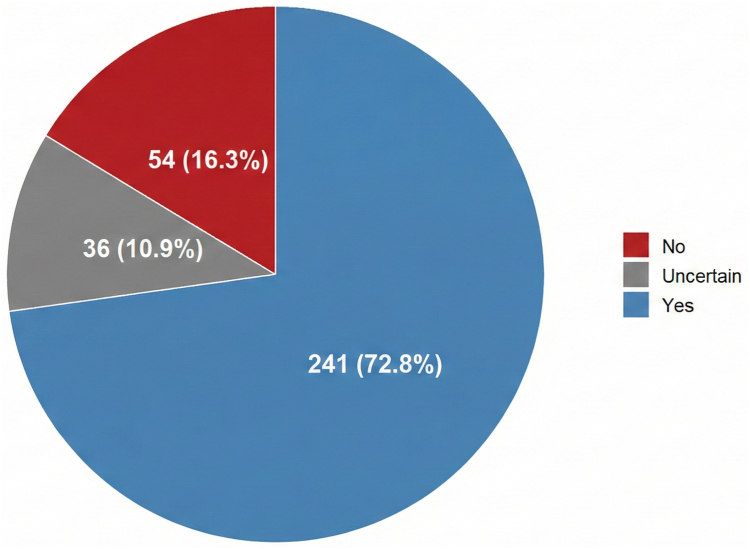
Instruction received to seek urgent care in case of venous thromboembolism symptoms. Numbers refer to pregnant individuals who received instruction for venous thromboembolism recognition.

In total, 331 (10.9%) respondents reported being instructed about the possible clinical manifestations of VTE, whereas 2383 (78.7%) reported not receiving any information (Figure 5). Among those who were instructed, 54 (16.3%) participants reported that they had not been advised to seek medical attention in case of suspected VTE (Figure 6).

**Figure 7:**
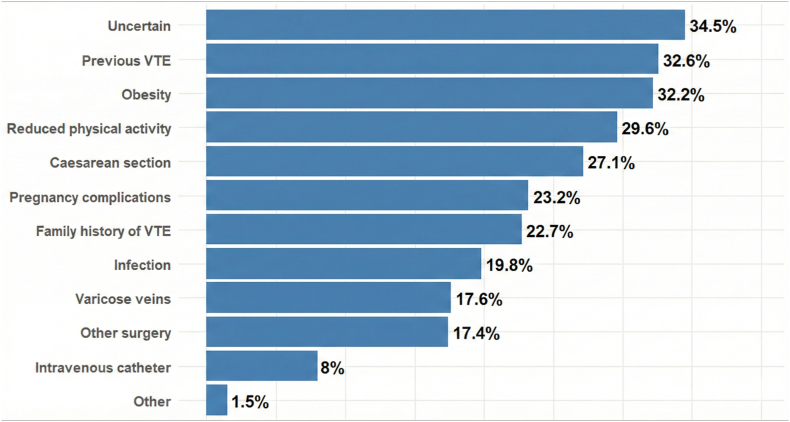
Participants’ knowledge of venous thromboembolism (VTE) risk factors.

**Figure 8:**
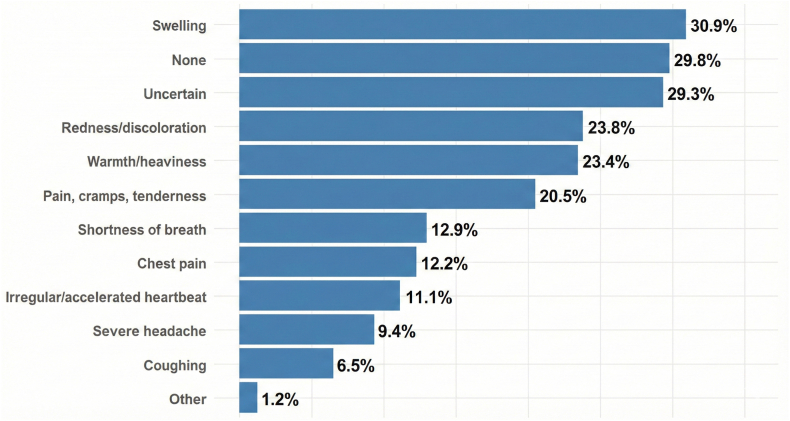
Participants’ knowledge of venous thromboembolism signs and symptoms.

The most frequently recognized risk factors for pregnancy-associated VTE were previous VTE (32.6%), obesity (32.2%), reduced physical activity (29.6%), and cesarean section (27.1%). As many as 34.5% of respondents indicated that they did not know any of the risk factors listed in the questionnaire (Figure 7). Regarding signs and symptoms potentially suggestive of DVT, responders most frequently identified limb swelling (30.9%), warmth or heaviness (24%), redness or discoloration (23.8%), and pain or tenderness (20.5%). In contrast, symptoms potentially indicative of PE, such as shortness of breath (12.9%), chest pain (1.2%), and irregular heartbeat (11.1%), were less well known (Figure 8).

### 3.3 Prior experience of VTE or anticoagulant drugs, and engagement in anticoagulation-related decisions

A previous VTE event was reported by 401 (13.3%) respondents. However, the site of thrombosis was unspecified in most cases (76.8%), with lower extremity DVT reported in 9.7%, followed by PE (7%) and DVT at other sites (5.7%), including splanchnic thrombosis (Table 3). When information about the timing of VTE was available, 12.7% of VTE cases occurred before pregnancy, 6% during pregnancy, and 5% after delivery.

**Table 3:** Participant-reported characteristics of venous thromboembolism, anticoagulation exposure, and perceived engagement in anticoagulant-related decisions.

Survey item	Survey participants (*N* = 3025), *n* (%)
Prior VTE	
Yes	401 (13.3)
Site of VTE^a^	
Lower extremity DVT	39 (9.7)
Upper extremity DVT	3 (0.7)
PE	28 (7.0)
Other sites	23 (5.7)
Not specified	308 (76.8)
Timing of thrombosis in relation to pregnancy status^a^	
Before pregnancy	51 (12.7)
During pregnancy	24 (6.0)
After delivery	20 (5.0)
Do not remember	16 (4.0)
Not specified	290 (72.3)
Self-reported actual or previous anticoagulation therapy and indication	
Yes	647 (21.4)
DVT before pregnancy	130 (4.3)
DVT during pregnancy or after delivery	243 (8.0)
Other reasons (prevention)	274 (9.1)
No	2090 (69.1)
Self-reported feelings about anticoagulation therapy	
Injections are perceived as extremely burdensome and distressing	100 (3.3)
Occasionally skipped injections due to discomfort with treatment	23 (0.7)
Would prefer an oral treatment, if available	255 (8.4)
No problems or distress with injections	199 (6.5)
Other	17 (0.6)
Uncertain	35 (1.2)
Self-reported use of aspirin and indication	
Yes	621 (20.5)
VTE prevention during pregnancy	421 (13.9)
Other	200 (6.6)
No	2129 (70.4)
Uncertain	148 (4.9)
Self-reported engagement in medical discussion regarding anticoagulant thromboprophylaxis during pregnancy	
Yes	542 (17.9)
No	2239 (74.0)
Uncertain	140 (3.4)
Informed about the risk of bleeding complications during anticoagulation and/or aspirin therapy	
Yes, thorough and clear information	237 (7.8)
Yes, scanty and/or unclear information	207 (6.8)
No	705 (23.3)
Self-reported worries about VTE risk and anticoagulation-associated complications	
Tension and/or anxiety	107 (3.5)
Depression and/or frustration	32 (1.0)
Other	12 (0.4)
No, neutral/unconcerned	433 (14.3)
Uncertain	62 (2.0)

DVT, deep vein thrombosis; PE, pulmonary embolism; VTE, venous thromboembolism.

a Values refer to responders who reported prior VTE (*n* = 401).

Overall, 21.4% of those surveyed reported anticoagulant use during their lifetime for either VTE treatment (12.3%) or other indications, including VTE prevention (9.1%).

Approximately half of the participants with current or past anticoagulant use reported no major difficulties with this therapy. A minority described injections as extremely burdensome and distressing (3.3%), while 0.7% admitted occasionally skipping injections due to discomfort. A preference for oral treatment was expressed by 8.4%. In addition, 621 (20.5%) respondents reported taking aspirin, either for VTE prevention during pregnancy (13.9%) or other reasons (6.6%).

As many as 74% of the surveyed pregnant individuals indicated that they did not engage with healthcare providers in discussions about the possibility of receiving anticoagulant prophylaxis for pregnancy-associated VTE before or during pregnancy or postpartum. Among respondents who had been involved in thromboprophylaxis discussions or had previously been exposed to anticoagulation, 32.4% remained unaware of the potential risk of bleeding complications associated with the use of these medications.

Among respondents who reported VTE education, almost 20% expressed psychological or emotional distress when becoming aware of the potential risks and implications connected with pregnancy-associated VTE (Supplementary Table S3).

### 3.4 Perceived relevance and participants’ experience with VTE education and awareness

More than two-thirds (74.4%) of the surveyed pregnant individuals believed that receiving education on pregnancy-associated VTE was essential or very important (Supplementary Figure S1). The proportion of respondents who rated VTE education as highly relevant was numerically higher among participants with higher levels of education attained (ie, university, PhD, or Master’s degree) and those aged 30 to 39 years (Supplementary Table S2). Respondents who conceived through assisted reproduction treatment also more frequently considered VTE education highly relevant compared with those who conceived naturally, while no clear difference was observed between pregnant individuals undergoing cesarean and vaginal delivery. When asked about their preferred method of receiving information, most respondents favored verbal communication (72.7%), followed by written booklets (45.3%). Digital formats, such as videos (22.6%) and applications (10.8%), were selected less frequently (Supplementary Figure S2).

## 4 Discussion

This multinational cross-sectional study provides valuable insights into pregnant individuals’ awareness and understanding of VTE and vascular health during pregnancy and the postpartum period. With more than 3000 participants, this is one of the largest studies to date, encompassing participants from 38 countries and diverse healthcare systems worldwide. Our findings reveal substantial unmet needs in education and awareness regarding pregnancy-related VTE.

Consistent with a recent global survey of patients living with cancer [22], which reported similarly poor knowledge and understanding of VTE risk and symptoms, our study found that, when restricting the analysis to respondents without a history of VTE, more than 70% reported receiving no or delayed VTE education during pregnancy or after delivery, underscoring the magnitude of the awareness gap in the general obstetric population.

These results highlight a concerning mismatch between the actual prevalence and severity of DVT and PE in pregnant individuals and the lack of awareness about the heightened thrombotic risk during pregnancy and the peripartum period. Such education and awareness gaps can lead to overlooked or misinterpreted symptoms, potentially resulting in delayed or even missed VTE diagnoses with delayed treatment and excess risk for serious and potentially fatal complications. As many symptoms are common in pregnancy, they are frequently attributed to the pregnancy itself rather than prompting suspicion of VTE, further contributing to diagnostic delay. Moreover, among participants who had received information, nearly two-thirds reported only a limited understanding of VTE, with unresolved questions or poor comprehension of the information delivered or the material provided, likely reflecting suboptimal or ineffective communication strategies.

**Figure 9:**
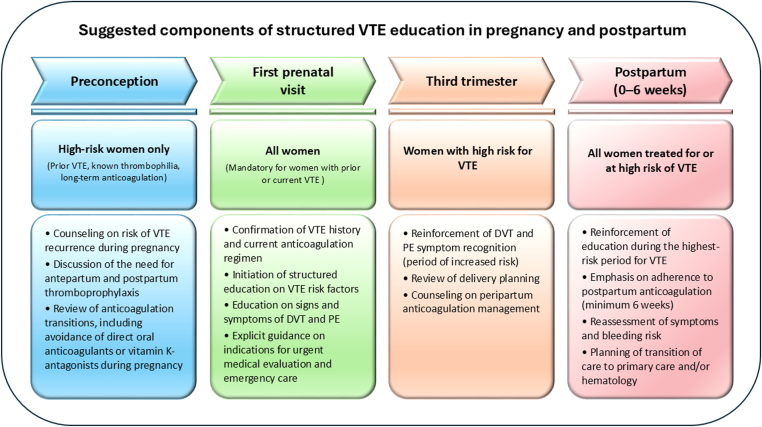
Proposed components of venous thromboembolism (VTE) education during pregnancy/postpartum. DVT, deep vein thrombosis; PE, pulmonary embolism.

The most commonly reported sources of VTE education were gynecologists or obstetricians, though other healthcare providers (eg, general practitioners and nurses) and nonmedical channels (social media and patient associations) were also identified. These data indicate that a multidirectional flow of information may effectively contribute to the education of pregnant individuals; however, they also highlight that substantial potential for further refinement and expansion remains. Moreover, these findings support the need for a structured, multidisciplinary education pathway with clear ownership. In practice, education should be obstetrician-led, integrated into routine antenatal care, and reinforced by midwives and primary care providers, with involvement of hematology or internal medicine when indicated (eg, prior VTE, thrombophilia, or complex anticoagulation decisions). Importantly, education should be delivered at defined time points: preconception counseling for individuals with a history of VTE or other high-risk features; a standardized discussion at the first prenatal visit for all pregnant individuals; and reinforcement in the third trimester and postpartum, when risk and clinical decision-making are particularly relevant. This approach moves beyond opportunistic counseling driven by clinical events and promotes equitable access to information (Figure 9).

Importantly, VTE education was not reported equally across different participant subgroups. Age, educational attainment, and the geographic or healthcare setting of origin did not appear to influence only the likelihood of receiving VTE education but also the related quality and level of understanding achieved. In addition, the need to undergo invasive procedures, such as assisted reproduction or cesarean delivery, seemed to be associated with a greater probability of receiving VTE-related counseling, possibly reflecting a higher perceived risk by healthcare providers in these contexts. These findings suggest that education of pregnant individuals concerning VTE is currently delivered in a selective and nonstandardized manner, shaped by clinical circumstances and sociodemographic factors rather than by shared principles of equitable and inclusive health communication.

A further knowledge gap emerged regarding the understanding of clinical manifestations and risk factors of VTE. Only 1 in 10 respondents had ever been instructed about VTE symptoms, and even among those informed, recognition of PE-related symptoms was particularly poor. This lack of awareness is likely to contribute to delays in diagnosis and treatment, with potential detrimental repercussions on maternal outcomes. Future educational materials should also address common venous manifestations, such as varicose veins, to help pregnant and postpartum individuals distinguish benign venous symptoms from features that warrant clinical assessment. Educational interventions have shown measurable benefits in other populations (eg, an educational video for patients living with cancer significantly reduced the time to VTE diagnosis and anticoagulation), suggesting that similar approaches may be effective in pregnancy, although they remain unexplored to date [23].

The use of thromboprophylaxis is often recommended for pregnant individuals with a history of unprovoked VTE or VTE associated with oral contraceptives or prior pregnancy, although recommendations vary substantially across guidelines [12] due to the limited evidence available.

Equally concerning is the low level of engagement in anticoagulation-related decision-making. In the present study, only 25% of respondents indicated discussing the option of primary thromboprophylaxis with their treating physicians, which may reflect the above-mentioned heterogeneity in international and national recommendations. Moreover, it is of concern that only one-third of individuals who had been exposed to anticoagulation in the past or at the time of survey completion were aware of the potential risk for bleeding complications. Among respondents who had received VTE education or engaged in anticoagulation-related discussions, nearly 20% continued to report emotional or psychological distress related to these issues. These findings suggest that education should not only focus on reducing the risk of thrombosis and associated complications through pharmacologic and nonpharmacologic strategies, but also aim to mitigate anxiety and concerns, thereby supporting pregnant individuals’ psychological well-being during pregnancy and the postpartum period.

Practical approaches may include framing risks in absolute numbers and plain language, emphasizing actionable “red-flag” symptoms and clear care pathways rather than alarmist messaging, and using tiered counseling that provides a concise core message for all patients, with the option of more detailed discussion for those at higher risk. Shared decision-making is particularly important when discussing thromboprophylaxis, with explicit discussion of expected benefits and bleeding risks, as well as opportunities for questions and follow-up. Finally, providing standardized written or digital materials from trusted sources that patients can revisit, and signposting to additional support when distress is identified, may help improve understanding while protecting psychological well-being.

Prior research on pregnancy-related VTE has primarily focused on women’s values and preferences regarding VTE thromboprophylaxis during pregnancy or the postpartum period, aiming to understand pregnant individuals’ perspectives on thromboprophylaxis use and the best ways to communicate the benefits and risks of heparin. Rodger et al. [24] explored pregnant individuals’ preferences for antepartum VTE thromboprophylaxis and found that females placed similar value on recurrent VTE and obstetric bleeding, although there was variability among participants. In another study involving 122 pregnant or postpartum individuals, most participants showed a preference for postpartum thromboprophylaxis over no treatment, even when the risk of postpartum VTE was low [25], in contrast to the previous study [24], where the acceptable threshold for agreeing to start antenatal thromboprophylaxis was higher.

Our study underscores the alarmingly low level of VTE education in the population at risk, with important implications for maternal health worldwide. These findings call for female-centered communication strategies and the integration of structured educational modalities into routine antenatal and postpartum care, not only to raise awareness and improve understanding, but also to mitigate anxiety and support quality of life.

Several limitations should be acknowledged. First, nearly half of the respondents were from Asia, and baseline rates of pregnancy-associated VTE are reported to be lower in Asian populations than in other regions. This demographic composition may therefore limit the generalizability of our findings to other populations and settings. Although our primary outcome focuses on education and awareness rather than VTE incidence, differences in baseline risk and healthcare pathways across regions may affect the perceived relevance of counseling and access to educational resources. In addition, awareness levels may differ across underrepresented regions and among individuals with lower educational attainment, and the relatively high proportion of respondents with university or advanced degrees may lead to overestimation of awareness in some settings. Second, the survey was distributed online rather than through systematic invitations, potentially leading to selection bias, particularly the underrepresentation of females with lower education or limited digital access, and the possible overrepresentation of females with prior VTE through thrombosis networks. Because the survey was disseminated via open online channels, the number of individuals who viewed the survey invitation is unknown, and a response rate cannot be calculated. Third, recall bias may have affected responses, and some variability across survey domains may reflect differences in comprehension of language and content. Fourth, subgroup comparisons were exploratory and were not adjusted for multiple testing; findings should be interpreted as hypothesis-generating. Finally, lower reported education rates among younger participants may partly reflect a lower absolute VTE risk at younger ages; however, this should not be considered a justification for omitting systematic education, which remains essential to support timely symptom recognition and informed decision-making. Despite these limitations, our survey represents the largest and most comprehensive international effort to date to capture pregnant individuals’ awareness and experiences regarding VTE in pregnancy and the postpartum period.

## 5 Conclusions

In conclusion, this large contemporary survey study enrolling more than 3000 females highlights the need to develop and implement standardized, shared, equitable, and inclusive strategies for VTE education during pregnancy, ensuring that pregnant individuals across diverse backgrounds and healthcare systems are equipped with the knowledge to recognize symptoms, engage in discussions about prophylaxis measures, and reduce the burden of pregnancy-associated VTE.
