# Supplementary material for: Global survey of women’s awareness of pregnancy- and postpartum-associated venous thromboembolism (World Thrombosis Day 2025)
Source: Res Pract Thromb Haemost. 2026 Mar 23;10(3):103421. doi: 10.1016/j.rpth.2026.103421 (PMC13099470; doi:10.1016/j.rpth.2026.103421)
Supplement: Supplementary Material [file mmc1.docx]

*Supplementary material*

1. **Supplementary Table S1 – Online Survey**

**Assessing awareness and education of women about the potential risks of blood clots during pregnancy and after delivery**

***A Global Initiative for the World Thrombosis Day 2024***

1. **Age:**

- ≤19
- 20-29
- 30-39
- ≥40

1. **Self-reported race/ethnicity:**

- Asian
- Black or African American
- Hispanic/Latino
- White
- Other, please specify: _________________
- Prefer not to say

1. **Country (please indicate from the list)**: ____________________________
2. **Education (please indicate highest degree):**

- None
- Primary school
- Secondary school
- College/University
- Advanced graduate/professional degree (for example PhD and/or Master)
- Other, please specify: _________________

1. **Have you become pregnant with the help of assisted reproduction treatment (for example: hormonal treatment, in-vitro fertilization, intracytoplasmic sperm injection, embryo transfer)?**

- No, I conceived naturally
- Yes, I have received assisted reproduction treatment
- I am uncertain/don’t know
- Prefer not to say

1. **Your pregnancy status:**

- I am less than 13 weeks pregnant
- I am 14-27 weeks pregnant
- I am 28-40 weeks pregnant
- I am more than 40 weeks pregnant
- I gave birth in the last 6-12 weeks
- Other, please specify: _____________________

1. **Have you undergone/will you undergo caesarean section?**

- Yes
- No, I have delivered/will deliver vaginally
- I am uncertain/don’t know yet

1. **Have you ever received information about the potential risk of thrombosis, that is the formation of blood clots in your veins and/or lungs, before pregnancy, during pregnancy or after delivery?**

- Yes
- No
- I am uncertain/don’t know

**8a) If yes, who gave you this information? (please select all that apply)**

- Gynecologist/obstetrician
- Internist
- Hematologist
- Surgeon
- Other specialist doctor
- Family doctor
- Nurse
- Other health-care professional
- Other (for example: family member or friend, experience from another patient, patient association, television, radio, social media, etc)

**8b) If yes, how would you rate your overall understanding of the information about risk of blood clots that you received?**

- Clear understanding with no questions
- Good understanding with few questions
- Medium understanding with many questions
- Unclear understanding and feeling confused

**8c) If yes, when did you first receive information about the risk of blood clots? (please select all that apply)**

- Before pregnancy
- At the time I was pregnant
- During pregnancy
- Before delivery
- After delivery
- At the time of blood clot diagnosis

1. **Which of the following do you consider would increase your risk of getting a blood clot during pregnancy or after delivery?** **(please select all that apply)**

- Caesarean section
- Other surgery
- Infection
- Obesity
- Pregnancy complications (for example: child small for stage of pregnancy, preeclampsia)
- Reduced level of physical activity or bedrest
- Previous blood clot
- Previous blood clot in a first-degree family member (parents or siblings)
- Catheter into the vein of the arm (for example to receive medications)
- Varicose veins
- Other, please specify: ___________________
- I am uncertain/don’t know

1. **Did your treating health care professional provide you with the information about the signs and symptoms of a blood clot during pregnancy or after delivery?**

- Yes
- No
- I am uncertain

**10a) If yes, have you been informed to get urgent medical care if you suspect you have a blood clot?**

- Yes
- No
- I am uncertain/don’t know

1. **Are you aware of any of the following signs and symptoms of a blood clot in pregnancy or after delivery? (please select all that apply)**

- Swelling of the arm, foot, ankle or leg on one side
- Redness or discoloration of the leg or arm
- Warmth or heaviness in the leg or arm
- Pain, cramping or tenderness in the calf or arm
- Shortness of breath
- Chest pain
- Coughing up blood
- Irregular or accelerated heartbeat/palpitations
- Very severe headache that won’t go away
- Other
- None
- I am uncertain/don’t know

**12) Have you ever been diagnosed with vein thrombosis (blood clot in the veins) or pulmonary embolism (blood clot in the lungs)?**

- Yes, vein thrombosis (blood clot) in the leg
- Yes, vein thrombosis (blood clot) in the arm
- Yes, vein thrombosis (blood clot) in other sites
- Yes, pulmonary embolism (blood clot in the lungs)
- No
- I am uncertain

**12a) If yes, when were you diagnosed with venous thrombosis?**

- Before pregnancy
- During pregnancy
- After delivery
- I do not remember

1. **Has any doctor ever discussed with you the possibility of starting anticoagulants (blood thinners) with injections (for example drugs called heparins/low molecular weight heparins including enoxaparin, tinzaparin, dalteparin, parnaparin etc) to prevent the formation of blood clots during pregnancy or after delivery?**

- Yes
- No
- I am uncertain/don’t know/don’t remember

**14) Have you used/are you using anticoagulants (blood thinners)?**

- Yes, I used/am using blood thinners for venous thrombosis (blood clot in the legs, lungs or other veins) that occurred **before** current pregnancy
- Yes, I am using blood thinners for venous thrombosis (blood clot in the legs, lungs or other veins) that occurred **during** current pregnancy or after delivery
- Yes, I have **previously** used blood thinners for other reasons (for example to prevent the blood clot in the legs or lungs)
- Yes, **I am using** blood thinners for other reasons (for example to prevent the blood clot in the legs or lungs)
- No, I have never used blood thinners
- I am uncertain/don’t know/don’t remember

**14a) (If yes to question 14), if you have received or are receiving blood thinners with subcutaneous injections (for example heparin) during your pregnancy or after delivery, how do you feel about this treatment? (Please select all that apply)**

- I find injections extremely burdensome and distressing
- I am very uncomfortable with this treatment and have spontaneously decided to skip a few injections
- I would prefer an oral treatment, if available
- I have no problems, pain or distress with injections
- Other, please specify: _________________________
- I am uncertain

**15) Have you used or are you using aspirin?**

- Yes, I have used/am using aspirin to prevent venous thrombosis (blood clot in the legs, lungs or other veins) during current pregnancy
- Yes, I have used/am using aspirin for other reasons (for example to prevent heart attack or stroke)
- No, I have never used aspirin
- I am uncertain

**16) (If yes to question 14 and/or 15): If you have used or are using anticoagulants (blood thinners) or aspirin, were you informed about the risk of bleeding complications (for example increased bruising or wound hematoma) associated with these medications?**

- Yes, I received thorough and clear information about the pro- and cons- of anticoagulants (blood thinners) or aspirin
- Yes, but I received scanty and/or unclear information about the pro- and cons- of anticoagulants (blood thinners) or aspirin
- No, I received no information about the risk of bleeding complications associated with blood thinners or aspirin

**17)(If yes to questions 8 or 16): After you received information about the risks of blood clots during pregnancy or after delivery, or of bleeding complications associated with blood thinners or aspirin, do you have particular worries (for example emotional or social)? (Please select all that apply)**

- Yes, I feel tense or anxious
- Yes, I feel depressed or frustrated
- Yes, other, please specify: ______________
- No, I feel neutral or unconcerned
- No, other, please specify: _______________
- I am uncertain

**18) Do you believe it was/is/would have been important to receive information about the risks, signs and symptoms of blood clots during pregnancy or after delivery?**

- Absolutely essential
- Very important
- Somewhat important
- Not very important
- I am uncertain

**19) What would be your most preferred method of receiving information about the risk, signs and symptoms of blood clots during pregnancy and after delivery? (Please select all that apply)**

- Verbally
- Written booklet
- Video
- App
- Poster in clinic
- Other, please specify: ________________

1. **Supplementary Table S2: Self-reported relevance of VTE awareness across participant subgroups.**

| **Characteristics** | **Relevance of VTE education n (%)** | | | | **P value** |
| --- | --- | --- | --- | --- | --- |
|  | **Highly relevant^a^** | **Moderately relevant** | **Scarcely relevant** | **Uncertain** |  |
| **Age (y)** | | | | | |
| < 19 | 21 (47.0) | 18 (40.9) | 1 (2.3) | 4 (9.1) | <.001 |
| 20-29 | 638 (72.6) | 180 (20.5) | 30 (3.4) | 30 (3.4) |  |
| 30-39 | 1390 (79.1) | 289 (16.4) | 32 (1.8) | 46 (2.6) |  |
| > 40 | 201 (75) | 52 (19.4) | 5 (1.9) | 10 (3.7) |  |
| **Self-reported ethnicity** | | | | | |
| Asian | 1068 (74.3) | 295 (20.5) | 23 (1.6) | 51 (3.5) | <.001 |
| Black or African American | 238 (78) | 51 (16.7) | 7 (2.3) | 9 (2.9) |  |
| Hispanic or Latino | 46 (88.4) | 5 (9.6) | 1 (1.9) | 0 (0) |  |
| White | 659 (79.9) | 125 (15.1) | 20 (2.4) | 21 (2.5) |  |
| Other | 60 (68.9) | 12 (13.8) | 9 (10.3) | 6 (6.9) |  |
| Prefer not to say | 178 (74.2) | 51 (21.2) | 8 (3.3) | 3 (1.2) |  |
| **Continent of residence** | | | | | |
| Africa | 23 (92) | 1 (4) | 0 (0) | 1 (4) | <.001 |
| The Americas | 25 (92.6) | 2 (7.4) | 0 (0) | 0 |  |
| Asia | 1064 (74.1) | 292 (20.3) | 21 (1.4) | 58 (4) |  |
| Europe | 1129 (77.9) | 242 (16.7) | 47 (3.2) | 31 (2.1) |  |
| Oceania | 9 (81.8) | 2 (18.1) | 0 (0) | 0 (0) |  |
| **Self-reported educational status** | | | | | |
| None | 34 (66.7) | 10 (19.6) | 4 (7.8) | 3 (5.9) | < .001 |
| Primary | 93 (56.4) | 55 (33.3) | 8 (4.85) | 9 (5.4) |  |
| Secondary | 495 (71.9) | 154 (22.4) | 12 (1.7) | 27 (3.9) |  |
| College | 357 (73.8) | 104 (21.5) | 13 (2.7) | 10 (2.1) |  |
| University | 607 (84.7) | 87 (12.1) | 5 (0.7) | 18 (2.5) |  |
| PhD or master | 589 (79.7) | 119 (16.1) | 18 (2.4) | 13 (1.8) |  |
| Other | 75 (72.8) | 10 (9.7) | 8 (7.8) | 10 (9.7) |  |
| **Method of conception** | | | | | |
| Naturally | 1930 (75.6) | 485 (19) | 60 (2.3) | 79 (3.1) | 0.008 |
| Assisted reproduction treatment | 263 (85.1) | 35 (11.3) | 4 (1.3) | 7 (2.3) |  |
| **Caesarean section** | | | | | |
| Yes | 1001 (76.3) | 248 (18.9) | 35 (2.7) | 28 (2.1) | < .001 |
| No (vaginal delivery) | 670 (77.8) | 146 (16.9) | 12 (1.4) | 33 (3.8) |  |

**^a^** includes the following survey response options: “absolutely essential” and “very important”

1. **Supplementary Table S3: Self-reported psychological impact associated with VTE awareness by participant subgroups.**

| **Characteristics** | **Psychological distress n (%)** | | | **P value** |
| --- | --- | --- | --- | --- |
|  | **Yes^a^** | **No** | **Uncertain** |  |
| **Age (y)** | | | | |
| < 19 | 0 (0) | 1 (100) | 0 (0) | 0.45 |
| 20-29 | 27 (17.8) | 107 (70.4) | 18 (11.8) |  |
| 30-39 | 88 (20.6) | 303 (71) | 36 (8.4) |  |
| > 40 | 22 (28.6) | 47 (61) | 8 (10.4) |  |
| **Self-reported ethnicity** | | | | |
| Asian | 48 (23.9) | 130 (64.7) | 23 (11.4) | 0.015 |
| Black or African American | 9 (12.5) | 54 (75) | 9 (12.5) |  |
| Hispanic or Latino | 6 (33.3) | 11 (61.1) | 1 (5.5) |  |
| White | 65 (21.8) | 209 (70.1) | 24 (8) |  |
| Other | 7 (41.2) | 9 (52.9) | 1 (5.8) |  |
| Prefer not to say | 2 (4) | 44 (88) | 4 (8) |  |
| **Continent of residence** | | | | |
| Africa | 1 (10) | 5 (50) | 4 (40) | <.001 |
| The Americas | 8 (50) | 6 (37.5) | 2 (12.5) |  |
| Asia | 47 (23.2) | 130 (64) | 26 (12.8) |  |
| Europe | 79 (18.8) | 313 (74.7) | 27 (6.5) |  |
| Oceania | 2 (22.2) | 4 (44.4) | 3 (33.3) |  |
| **Self-reported educational status** | | | | |
| None | 2 (28.5) | 5 (71.4) | 0 (0) | 0.288 |
| Primary | 2 (16.6) | 9 (75) | 1 (8.3) |  |
| Secondary | 31 (27.2) | 66 (57.9) | 17 (14.9) |  |
| College | 19 (20) | 67 (70.5) | 9 (9.5) |  |
| University | 40 (21.5) | 128 (68.8) | 18 (9.7) |  |
| PhD or master | 37 (17.1) | 165 (76.4) | 14 (6.5) |  |
| Other | 6 (22.2) | 18 (66.7) | 3 (11.1) |  |
| **Method of conception** | | | | |
| Naturally | 106 (19.9) | 374 (70.2) | 53 (9.9) | 0.224 |
| Assisted reproduction treatment | 29 (26.4) | 75 (68.2) | 6 (5.5) |  |
| **Caesarean section** | | | | |
| Yes | 51 (20.3) | 172 (68.5) | 28 (11.1) | 0.248 |
| No (vaginal delivery) | 46 (18.2) | 186 (73.5) | 21 (8.3) |  |

^a^includes the following survey response options: “tense or anxious”, “depressed or frustrated” and any other form of psychological distressed reported (“other”)

**Supplementary Figure S1: Perceived importance of VTE education**

**
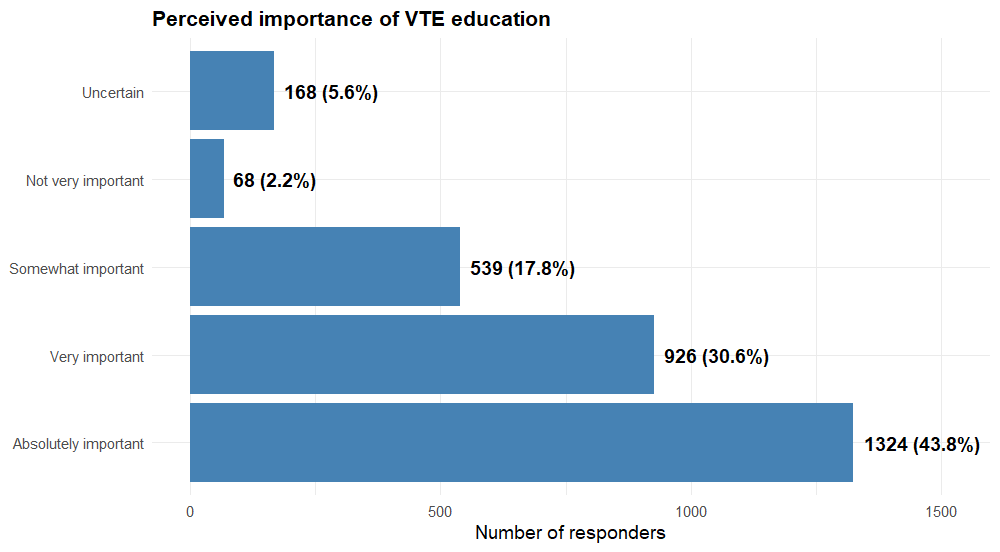
**

1. **Supplementary Figure S2: Preferred method of receiving VTE education**

**
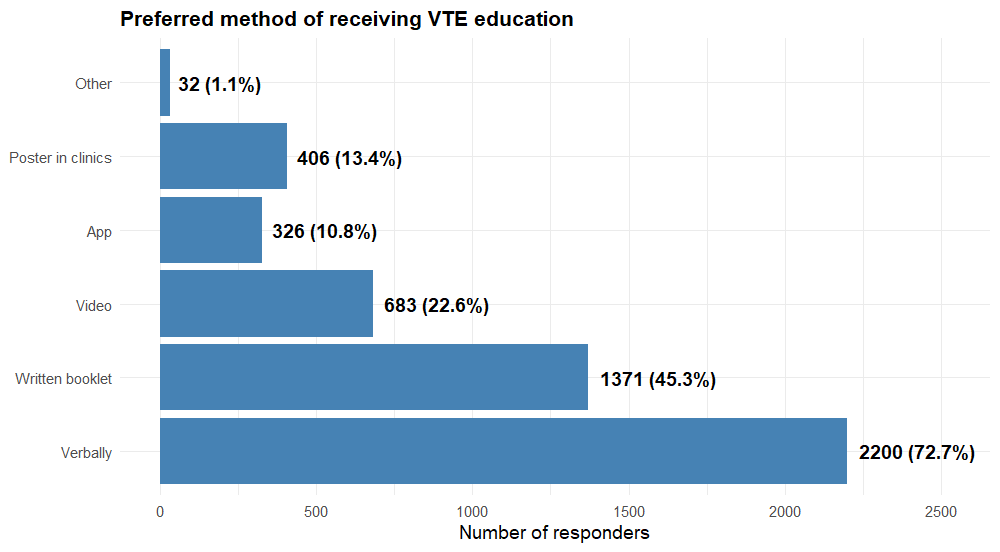
**
